# Protein Stability—Analysis of Heat and Cold Denaturation without and with Unfolding Models

**DOI:** 10.1021/acs.jpcb.3c00882

**Published:** 2023-04-11

**Authors:** Joachim Seelig, Anna Seelig

**Affiliations:** Biozentrum, University of Basel, Spitalstrasse 41, CH-4056 Basel, Switzerland

## Abstract

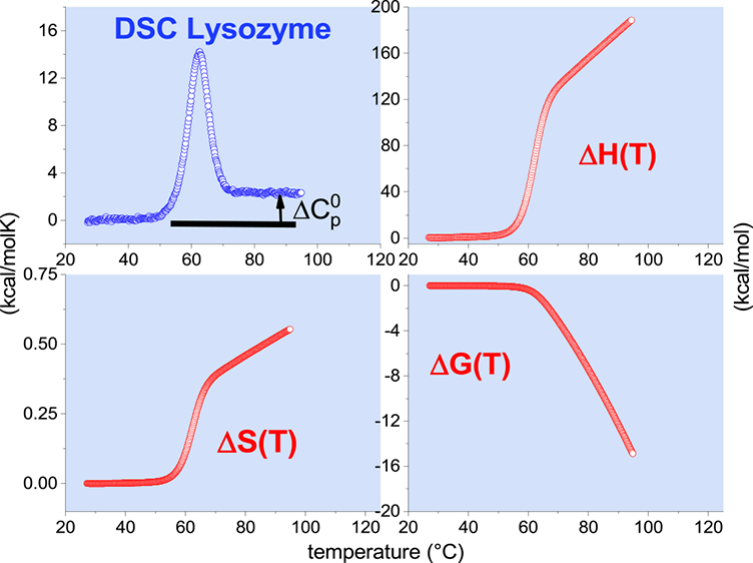

Protein stability is important in many areas of life sciences. Thermal protein unfolding is investigated extensively with various spectroscopic techniques. The extraction of thermodynamic properties from these measurements requires the application of models. Differential scanning calorimetry (DSC) is less common, but is unique as it measures directly a thermodynamic property, that is, the heat capacity *C*_p_(*T*). The analysis of *C*_p_(*T*) is usually performed with the chemical equilibrium two-state model. This is not necessary and leads to incorrect thermodynamic consequences. Here we demonstrate a straightforward model-independent evaluation of heat capacity experiments in terms of protein unfolding enthalpy Δ*H*(*T*), entropy Δ*S*(*T*), and free energy Δ*G*(*T*)). This now allows the comparison of the experimental thermodynamic data with the predictions of different models. We critically examined the standard chemical equilibrium two-state model, which predicts a positive free energy for the native protein, and diverges distinctly from the experimental temperature profiles. We propose two new models which are equally applicable to spectroscopy and calorimetry. The Θ_U_(*T*)-weighted chemical equilibrium model and the statistical-mechanical two-state model provide excellent fits of the experimental data. They predict sigmoidal temperature profiles for enthalpy and entropy, and a trapezoidal temperature profile for the free energy. This is illustrated with experimental examples for heat and cold denaturation of lysozyme and β-lactoglobulin. We then show that the free energy is not a good criterion to judge protein stability. More useful parameters are discussed, including protein cooperativity. The new parameters are embedded in a well-defined thermodynamic context and are amenable to molecular dynamics calculations.

## Introduction

Many proteins can be denatured by heating or cooling. The detailed knowledge of protein stability is thus an important problem in developing biological therapeutics. A large variety of spectroscopic methods is used to characterize protein unfolding. All these methods reflect structural changes. Their thermodynamic analysis requires the application of models without guaranteeing a correct image of the unfolding thermodynamics. In contrast, the thermodynamic properties of protein unfolding follow directly from the measurement of the heat capacity *C*_p_(*T*), to which spectroscopic results should then be compared.^1^

Here we demonstrate that differential scanning calorimetry (DSC) is the method of choice in analyzing the thermodynamic stability of proteins. The first modern DSC instruments were built independently by Brandts^2^ and by Privalov^3^ in the 1970s. The heat capacity was found to display a distinct maximum at the midpoint of unfolding. Surprisingly, the scientific interest remained focused on the model-dependent simulation of the heat capacity peak only. Further consequences with respect to entropy and free energy were not considered. We now demonstrate that a simple and model-independent analysis of heat capacity measurements provides all relevant thermodynamic properties of protein stability. Sigmoidal temperature-profiles are observed for enthalpy and entropy. Due to enthalpy-entropy compensation, the free energy of protein unfolding is small and displays a trapezoidal temperature profile. These model-independent thermodynamic results are used to compare different unfolding models.

Protein unfolding is a cooperative process with many short-lived intermediates. An important co-operative model has been published in 1959, but has largely been ignored.^4^ Instead, a chemical equilibrium two-state model has been proposed for small proteins that has dominated protein unfolding^2,5−13^ for the last 40 years. A two-state model considers only two types of protein conformations in solution, the native protein (N) and the fully unfolded protein (U). Here we compare calorimetric results of heat and cold denaturation of lysozyme and β-lactoglobulin with the predictions of different unfolding models. The standard chemical equilibrium two-state model makes incorrect predictions when compared to the experimental results. We therefore introduce two new two-state models. In particular, a statistical-mechanical two-state model yields excellent fits to all observed thermodynamic properties. A modified chemical equilibrium two-state model is also useful for most practical purposes. The new models are equally applicable to calorimetry and spectroscopy. Finally, thermodynamic criteria for protein stability are discussed. Cooperativity appears to be a better indicator of protein stability than changes in free energy.

## Methods

Published protein unfolding data, obtained with differential scanning calorimetry (DSC), are evaluated model-independently in terms of enthalpy, entropy, and free energy by standard thermodynamic methods. The experimental results are then compared to the predictions of two chemical equilibrium two-state models and a statistical model. The focus of the analysis is on the protein unfolding transition proper.

### Differential Scanning Calorimetry

“Differential scanning calorimetry (DSC) is a very powerful tool for investigating protein folding and stability because its experimental output reflects the energetics of all conformations that become minimally populated during thermal unfolding.”^8^ In a DSC experiment a sample cell contains the protein solution and a reference cell contains the same buffer. The difference in the amount of heat required to increase the temperature of sample and reference is measured as a function of temperature. Sample and reference are maintained at nearly the same temperature throughout the experiment. DSC allows a precise measurement of the heat capacity *C*_p_(*T*). In DSC unfolding experiments the protein heat capacity starts almost horizontally reflecting the basic heat capacity of the native protein.^14^ Upon unfolding, the heat capacity gives rise to a large heat peak. After unfolding, *C*_p_(*T*) displays again a smooth increase. Due to the additional binding of water molecules to the backbone and side chains of the unfolded protein, the heat capacity of the unfolded protein is larger than that of the native protein.^15^

DSC measurements with modern instruments are straightforward. An excellent review on the use of DSC in protein unfolding has recently been published by Ibarra-Molero et al.^8^ Here, we discuss aspects not included in this review, focusing on the unfolding transition proper. We use published DSC results where the basic heat capacity of the native protein was removed by appropriate baseline correction (for details see ref (8)). Hence the native protein has an apparent zero heat capacity. This is without loss of generality as was demonstrated previously.^16^

### Model-Independent Evaluation of the Heat Capacity *C*_p_(*T*) with Respect to Enthalpy, Entropy, and Free Energy

According to standard thermodynamics the DSC-measured heat capacity *C*_p_(*T*) is the derivative of the enthalpy *H*(*T*) at constant pressure *p*.
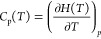
1

The precise measurement of the temperature profile of the heat capacity *C*_p_(*T*) provides the thermodynamic functions enthalpy, entropy, and Gibbs free energy. These properties of protein unfolding can be derived model-independently by numerical integration of the standard relations for enthalpy *H*(*T*) = ∫ *C*_p_(*T*)d*T*, entropy  and Gibbs free energy *G*(*T*) = *H*(*T*) – *TS*(*T*). In the DSC experiment the heat capacity is sampled in discrete temperature intervals Δ*T* and the above integrals can be evaluated as follows:^16^
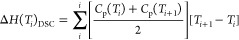
2
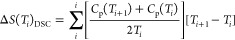
3
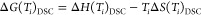
4These equations define the change of the thermodynamic functions in discrete temperatures steps as will be illustrated in more detail below. Equations 2–4 are of general validity and can also be applied to DSC thermograms, which are not baseline-corrected (see ref (16)).

In many published DSC experiments the native and the unfolded protein have the same zero heat capacity.^17^ The heat capacity difference Δ*C*_p_^0^ between native and unfolded protein is removed by baseline correction. This is unfortunate as “in considering the energetic characteristics of protein unfolding one has to take into account all energy which is accumulated upon heating, [...] that is, all the excess heat effects must be integrated″.^18^ The present analysis of experimental data always includes the increased heat capacity Δ*C*_p_^0^.

### Models for Protein Unfolding

Protein folding is a conformational reorganization involving the cooperation of many weak local contacts. The concept of ″downhill folding″ assumes that free energy barriers between protein-like states are intrinsically small^19,20^ in the funnel hypothesis pursued in molecular dynamics calculations. The native protein sits at the bottom of the funnel, which is a minimum of the free energy.

#### Standard Chemical Equilibrium Two-State Model

The chemical equilibrium two-state model is the long-standing model to analyze calorimetric (DSC) and spectroscopic protein unfolding transitions. It provides the van’t Hoff enthalpy of the N ⇄ U two-state equilibrium. The model assumes a temperature-dependent equilibrium between a single native protein (N) and a single denatured molecule (U).
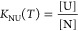
5

As the model is well-described (e.g., refs (12, 13)) we state the essential thermodynamic equations without further explanation
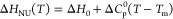
6
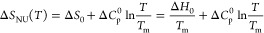
7
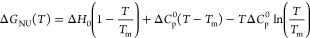
8

Identical equations in a more complex notation are found in review.^6^

Δ*H*_0_ is the conformational enthalpy (van’t Hoff enthalpy), Δ*C*_p_^0^ = *C*_p, end_ – *C*_p, ini_ is the heat capacity difference between the native and the unfolded protein. *T*_m_ is the midpoint temperature of unfolding.

**Figure 1:**
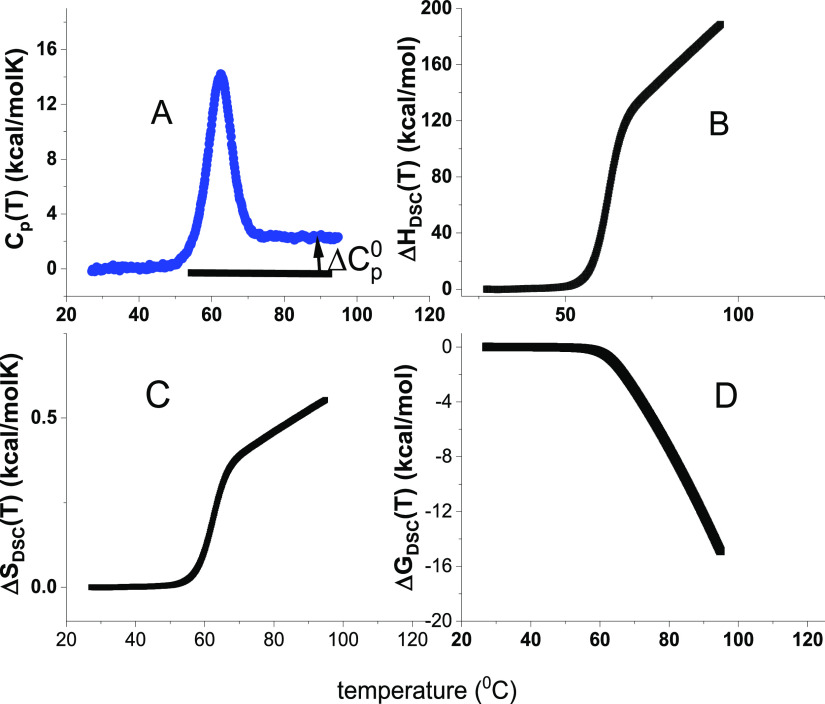
Model-independent evaluation of the molar heat capacity *C*_p_(*T*) of lysozyme. (A) Primary experimental data. Heat capacity *C*_p_(*T*) (50 μM lysozyme, 20% glycine buffer, pH 2.5). DSC data (temperature resolution 0.17 °C) taken from ref (1, 26). (B) Enthalpy Δ*H*(*T*)_DSC_ (eq 2). (C) Entropy Δ*S*(*T*)_DSC_ (eq 3). (D) Gibbs free energy Δ*G*(*T*)_DSC_ (eq 4).

Equations 6–8 ignore the large heat capacity peak of the unfolding reaction at *T*_m_ (see Figure 1A). Differential scanning calorimetry shows that the heat capacity of unfolding is a non-linear function of temperature with a pronounced *C*_p_(*T*)-maximum at *T*_m_. Consequently, the enthalpy Δ*H*(*T*) = ∫*C*_p_(*T*)d*T* and the entropy  are also non-linear functions of temperature. However, in contrast to the experimental observations, eqs 6 and 7 are linear or nearly linear functions.

Equation 8 defines the two-state equilibrium constant
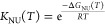
9and, in turn, the extent of unfolding
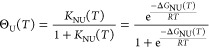
10

Equation 10 has a sigmoidal shape and is used to fit spectroscopic unfolding transitions. The model has some puzzling consequences. At the midpoint temperature *T*_m_ the model predicts Δ*G*_NU_(*T*_m_) = 0 and Θ_U_(*T*_m_) = 1/2. Even though only 50% of the protein is unfolded, eqs 6 and 7 predict 100% enthalpy Δ*H*_0_ and 100% entropy Δ*S*_0_. Another surprise is the positive free energy of the native protein (see Figure 1 in refs^12,13^). This is against the idea that the native protein constitutes a minimum of the free energy.

The calculation of the heat capacity requires an empirical extension of eq 6, according to Δ*H*_NU_(*T*)Θ_U_(*T*). The heat capacity is then given by
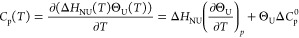
11

Equation 11 is identical to eq 14 in ref (6). It provides a good fit of the heat capacity curve of small proteins. However, eq 11 leads to another thermodynamic inconsistency. It predicts a zero heat capacity for the native protein as Θ_U_ = 0, which is contradicted by nonzero values for enthalpy, entropy and free energy at the same temperature (eqs 6–8). In contrast, DSC confirms zero values of all thermodynamic properties if the heat capacity is zero (see Figure 1).

#### Θ_U_(*T*)-Weighted Chemical Equilibrium Two-State Model

This model is a simple extension of the standard model by multiplying eqs 5–7 with the extent of unfolding Θ_U_(*T*) (eq 10) resulting in three new functions
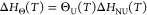
12
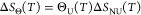
13
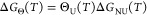
14

The heat capacity is given by eq 11. Equations 11–14 define the Θ_U_(*T*)-weighted chemical equilibrium *two*-state model, which has not yet been discussed in the relevant literature.

#### Partition Function

The heat capacity and other thermodynamic properties of protein unfolding are intimately related to the protein partition function *Z*(*T*) according to^21,22^
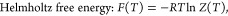
15
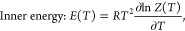
16
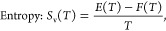
17
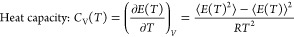
18

Equations 15–18 refer to reactions at constant volume. Volume changes in protein unfolding are rather small (≤5%).^23^ Hence the following identities hold: Δ*E* ≅ Δ*H*, Δ*S*_v_ ≅ Δ*S*_p_, Δ*F* ≅ Δ*G*.

#### Statistical-Mechanical Two-State Model. Macroscopic Parameters

We present a simple statistical-mechanical two-state model as an alternative to the chemical equilibrium two-state model. Based on the statistics of the linear Ising model^24^ as described in ref (25) the following continuous canonical partition function can be defined^26^
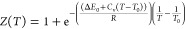
19

Δ*E*_0_ is the difference in inner energy between the native and the unfolded protein. Δ*E*_0_ is virtually identical to the conformational enthalpy Δ*H*_0_ as will be shown experimentally below. The inner energy Δ*E*_0_ is temperature-dependent with the heat capacity *C*_v_, which accounts for the increase Δ*C*_p_^0^ between the native and the denatured protein. The partition function *Z*(*T*) predicts all thermodynamic properties, in combination with eqs 15–18. The extent of unfolding is not needed in the calculation of thermodynamic properties and is given here for completeness only
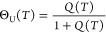
20with 

The statistical-mechanical two-state model provides an analytical expression for the temperature of cold denaturation. The midpoint of unfolding is
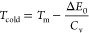
21Δ*E*_0_ and *C*_v_ have opposite effects on *T*_cold_. Δ*E*_0_ stabilizes the protein and lowers *T*_cold_, *C*_v_ represents energy fluctuations (eq 19), destabilizing the structure and increasing *T*_cold_.

#### Multistate Cooperative Unfolding Model. Molecular Parameters

The partition function determines the thermodynamic properties of the system (eqs 23–26).^21,22^ We use the partition function of the multistate cooperative Zimm–Bragg theory.^4,27,28^ The Zimm–Bragg theory has been applied successfully to the unfolding of helical and globular proteins of different structure and size.^1,16,26,29−34^ Here we use^16^
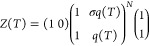
22
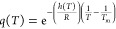
23
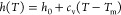
24*h*_0_ is the energy change of unfolding a single amino acid. *h*_0_ is temperature-dependent with heat capacity *c*_v_. *N* is the number of amino acids participating in the transition. The cooperativity parameter σ determines the sharpness of the transition. The smaller σ, the sharper is the transition. σ is typically 10^–3^–10^–6^. Equation 22 can be applied to proteins of any size, even antibodies with unfolding enthalpies of ∼1000 kcal/mol.^16^ In contrast, two-state models are best suited for small proteins with enthalpies of 50–200 kcal/mol.

## Results

### Lysozyme Heat Unfolding: Thermodynamic Parameters Obtained Model-Independently by DSC

Lysozyme (14.3 kDa) is a globular 129-residue protein with ∼25% α-helix, ∼40% β-structure and ∼35% random coil in solution at room temperature.^1^ Upon unfolding, the α-helix is almost completely lost and the random coil content increases to ∼60%. The DSC thermogram of lysozyme unfolding is shown in Figure 1. The baseline-corrected heat capacity Δ*C*_p_(*T*) of the native protein is zero (for detail see ref (16)), then goes through a maximum at the midpoint temperature *T*_m_ = 62 °C and levels off again. The heat capacity increases upon unfolding by Δ*C*_p_^0^ = 2.27 kcal/molK. (Literature: 1.54–2.27 kcal/molK^1,7,15,35−38^). The enthalpy Δ*H*(*T*)_DSC_ and entropy Δ*S*(*T*)_DSC_ are evaluated model-independently with eqs 2 and 3 and have sigmoidal shapes (Figure 1B,C). The free energy Δ*G*(*T*)_DSC_ (eq 4) of the native protein is zero, is slightly negative in the initial phase of unfolding, and decreases rapidly beyond the midpoint temperature *T*_m_ = 62 °C (Figure 1D).

The total unfolding enthalpy is Δ*H*_DSC_ = 138 kcal/mol. It is composed of the conformational enthalpy proper, Δ*H*_0_ and a contribution Δ*H*_Δ*C*_p_^0^_ caused by the heat capacity term Δ*C*_p_^0^.
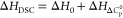
25

The two enthalpies can be separated by applying the models described above. In the model-independent analysis, the contribution Δ*H*_Δ*C*_p_^0^_ can be approximated as follows (eq 26). Equation 26 calculates the area of the triangle defined by the baseline *c*_end_ – *c*_ini_ and the height Δ*C*_p_^0^. The hypotenuse is a sigmoidal line which explains the factor 3 instead of 2 in the denominator.
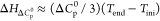
26The Δ*H*_Δ*C*_p_^0^_ values are confirmed by a comparison with the predictions of the Θ_U_(*T*)-weighted chemical equilibrium model or the statistical-mechanical models. For lysozyme with Δ*C*_p_^0^ = 2.269 kcal/mol K, *T*_ini_ = 318 K, and *T*_end_ = 346 K this results in Δ*H*_Δ*C*_p_^0^_ = 21.2 kcal/mol (simulations yield 20–24 kcal/mol). The experimental data for lysozyme are summarized in Table 1A.

Of note, ″unfolded″ proteins are not completely unfolded, but contain residual structure.^39,40^ Complete unfolding is difficult to achieve as many different physical and chemical factors contribute to protein stability.^40^ In the present evaluation the extent of unfolding is always Θ_U_ > 0.9 as judged by applying the unfolding models.

### β-Lactoglobulin Cold Denaturation—Thermodynamic Parameters Obtained Model-Independently by DSC

**Figure 2:**
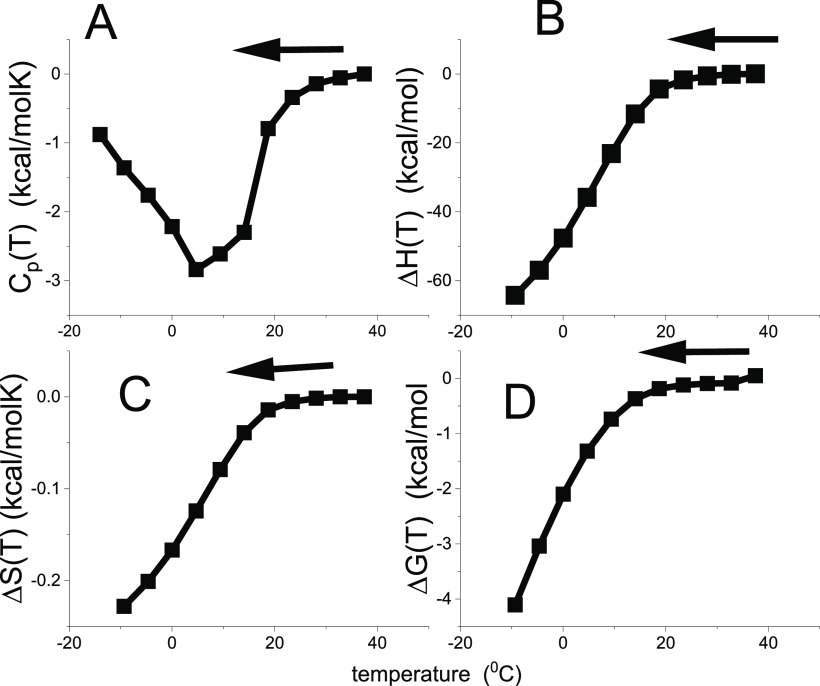
Cold denaturation of β-lactoglobulin. Data in panel A are taken from ref (41), Figure 2, 4 M urea. The arrows indicate the cooling direction. The DSC experiment starts with the native protein at ∼35 °C and the temperature is reduced linearly to −14 °C. The heat capacity of the native protein is zero due to baseline correction. (A) Heat capacity *C*_p_(*T*). (B) Enthalpy Δ*H*_DSC_(*T*). (C) Entropy Δ*S*_DSC_(*T*). (D) Gibbs free energy Δ*G*_DSC_(*T*).

DSC data for cold denaturation are scarce. One of the best examples is the unfolding of β-lactoglobulin in urea solution.^41^ Bovine β-Lactoglobulin is an 18.4 kDa protein comprising 162 amino acids that fold up into an 8-stranded, antiparallel β-barrel with a 3-turn α-helix on the outer surface. A DSC cold-denaturation experiment of β-lactoglobulin is shown in Figure 2 (data taken from ref (41)). The experiment starts with the native protein at ∼35 °C and the temperature is lowered gradually to −14 °C. The heat capacity of the native protein is zero and all thermodynamic functions are necessarily also zero at ambient temperature.

**Table 1:** (A–C) DSC Unfolding of Lysozyme and β-Lactoglobulin

parameters	units	DSC	Θ_U_(*T*)-weighted model	statistical-mechanical model
(A) Thermodynamic Parameter for Lysozyme Heat Unfolding				
*T*_ini_^a^	°C (K)	45 (318)	45	45
*T*_m_^a^	°C (K)	62 (335)	62	62
*T*_end_^a^	°C (K)	73 (346)	73	73
Δ*C*_p_^0^, Δ*C*_v_^b^	kcal/molK	2.27	2.27	1.05
Δ*H*_DSC_, Δ*H*_Θtotal_, Δ*E*_total_^c^	kcal/mol	137	130.7	132.3
Δ*H*_0_, Δ*E*_0_^d^	kcal/mol		107	110
Δ*H*_Δ*C*_p_^0^_^e^	kcal/mol	21.2	23.7	22.3
Δ*S*_DSC_, Δ*S*_p_, Δ*S*_v_^f^	kcal/molK	0.409	0.389	0.394
Δ*G*_DSC_, Δ*G*_0_, Δ*F*_0_^g^	kcal/mol	–4.27	–3.78	–3.87
Δ*H*_total_/Δ*S*_total_^h^	°C (K)	62 (335)	63 (336)	63 (336)
Δ*T*^i^	°C	n.d.	88	105
(B) Thermodynamic Parameter for β-Lactoglobulin Cold Unfolding				
*T*_ini_^a^	°C (K)	37 (310)	37	37
*T*_m_^a^	°C (K)	6 (279)	7	6
*T*_end_^a^	°C (K)	–14 (259)	–15	–15
Δ*C*_p_^0^, Δ*C*_v_^b^	kcal/molK	0.86	1.1	0.45
Δ*H*_DSC_, Δ*H*_Θtotal_, Δ*E*_total_^c^	kcal/mol	–69.5	–65	–59.2
Δ*H*_0_, Δ*E*_0_^d^	kcal/mol		–42	–42
Δ*H*_Δ*C*_p_^0^_^e^	kcal/mol	–18.9	–23.1	–17.2
Δ*S*_DSC_, Δ*S*_p_, Δ*S*_v_^f^	kcal/molK	–0.248	–0.235	–0.215
Δ*G*_DSC_, Δ*G*_0_, Δ*F*_0_^g^	kcal/mol	–4.1	–4.07	–3.65
Δ*H*_total_/Δ*S*_total_^h^	°C (K)	8 (280)	3 (276)	3 (276)
(C) Thermodynamic Parameter for β-Lactoglobulin Heat and Cold Unfolding				
*T*_ini_^a^	°C (°K)	28 (301)	28	28 (301)
*T*_m_^a^	°C (°K)	58 (331)	53 (326)	55 (328)
*T*_end_^a^	°C (°K)	71 (344)	71	71 (344)
Δ*C*_p_^0^, Δ*C*_v_^b^	kcal/molK	3.1	2.4	1.25
Δ*H*_DSC_, Δ*H*_Θtotal_, Δ*E*_total_^c^	kcal/mol	109	99.1	101
nd, Δ*H*_0_, Δ*E*_0_^d^	kcal/mol		56	60
Δ*H*_Δ*C*_p_^0^_^e^	kcal/mol	44.3	43	41
Δ*S*_DSC_, Δ*S*_p_, Δ*S*_v_^f^	kcal/molK	0.329	0.286	0.304
Δ*G*_DSC_, Δ*G*_0_, Δ*F*_0_^g^	kcal/mol	–4.12	–4.33	–3.89
Δ*H*_total_/Δ*S*_total_^h^	K	58 (331)	74 (347)	59 (332)
Δ*T*^i^	°C		47	48
*T*_ini_^a^	°C (K)	28 (301)	28 (301)	28 (301)
*T*_mcold_^a^	°C (K)	4 (277)	4 (277)	4 (277)
*T*_endcold_^a^	°C (K)	–7 (266)	–7 (266)	–7 (266)
Δ*C*_p_^0^, Δ*C*_v_^b^	kcal/molK	2.79	2.4	1.25
Δ*H*_DSC_, Δ*H*_Θtotal_, Δ*E*_total_^c^	kcal/mol	81.1	87.7	80
Δ*H*_0_, Δ*E*_0_^d^	kcal/mol	56	56	60
Δ*S*_DSC_, Δ*S*_p_, Δ*S*_v_^f^	kcal/molK	0.293	0.331	0.288
Δ*G*_DSC_, Δ*G*_0_, Δ*F*_0_^g^	kcal/mol	–3.06	–4.32	–3.29
Δ*H*_total_/Δ*S*_total_^h^	°C (K)	4 (277)	–8 (265)	5 (278)

a *T*_ini_, *T*_m_, *T*_end_: temperatures of beginning, midpoint, and end of protein unfolding.

b Δ*C*_p_^0^: total heat capacity change upon unfolding. Δ*C*_v_: heat capacity change of the inner energy *E*_0_.

c Δ*H*_DSC_: total enthalpy change measured with DSC between *T*_ini_ and *T*_end_. Δ*H*_Θtotal_, Δ*E*_total_: total heat of unfolding calculated with either the chemical equilibrium model or the statistical-mechanical model.

d Δ*H*_0_: conformational enthalpy change. Δ*E*_0_: conformational inner energy change.

e Contribution of the heat capacity Δ*C*_p_^0^ to the total unfolding enthalpy/energy.

f Δ*S*_DSC_: total entropy change measured with DSC between *T*_ini_ and *T*_end_. Δ*S*_p_, Δ*S*_v_: total entropy of unfolding calculated with either the chemical equilibrium model or the statistical-mechanical model.

g Δ*G*_DSC_: total free energy change measured with DSC between *T*_ini_ and *T*_end_. Δ*G*_0_, Δ*F*_0_: total free energy of unfolding calculated with either the chemical equilibrium model or the statistical-mechanical model.

h Prediction of the midpoint of unfolding as the ratio of measured or calculated total enthalpy and total entropy.

i Predicted temperature difference between heat to cold denaturation calculated with  (Θ_U_(*T*)-weighted chemical model) and Δ*T* = Δ*E*/*C*_v_ (statistical-mechanical model).

Cold denaturation is an exothermic reaction. At the end of the DSC experiment at −14 °C the released heat as evaluated with eq 1 is Δ*H*_DSC_ = −69.5 kcal/mol (−291 kJ/mol, in agreement with Table 2 in ref (41)). The corresponding entropy change is Δ*S*_DSC_ = −0.248 kcal/mol. The ratio Δ*H*_DSC_/Δ*S*_DSC_ = 280 K = 7 °C is close to the experimental *C*_p_(*T*) minimum at 277 K.

### β-Lactoglobulin. Heat-Induced Folding and Unfolding. Thermodynamic Parameters Obtained Model-Independently by DSC

**Figure 3:**
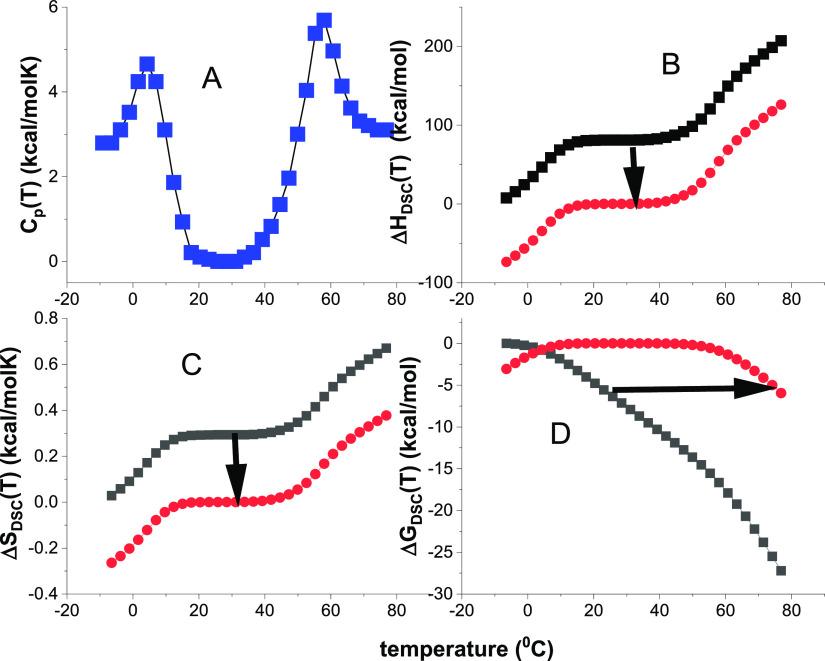
Heat-induced folding (at 4 °C) and unfolding (at 57 °C) of β-lactoglobulin in 2.0 M urea. Model-independent evaluation of the heat capacity *C*_p_(*T*). (A) DSC data taken from ref (41). (B) Enthalpy Δ*H*_DSC_(*T*). Black data points: integration of *C*_p_(*T*) according to eq 2. Red data points: black data points shifted downwards by 78.3 kcal/mol, the enthalpy of cold denaturation. (C) Entropy Δ*S*(*T*). Black: integration of *C*_p_(*T*) with eq 3. Red: primary data points shifted downwards by 0.283 kcal/molK, the unfolding entropy of cold denaturation. (D) Gibbs free energy Δ*G*_DSC_(*T*). Black: application of eq 4 to black data points in panels B and C. Red: combination of red data points in panels B and C, according to eq 4.

The DSC experiment shown in Figure 3 is unusual as it involves a disorder → order transition at low temperature and the reverse order → disorder transition at high temperature.

Figure 3A reports the DSC experiment. The heat capacity *C*_p_(*T*) is used to calculate the thermodynamic properties in Figure 3B–D. At the beginning of the DSC experiment at −9 °C, the protein is cold-denaturated and disordered. Upon heating, the protein goes through a disorder → order transition with a heat uptake of Δ*H*_DSC_ = 78.3 kcal/mol. At 25 °C the protein is in an ordered, native-like structure. Further heating induces new disorder with an enthalpy uptake of Δ*H*_DSC_ = 104.1 kcal/mol. *C*_p_(*T*) shows maxima at 4 and 57 °C. The entropies increase by Δ*S*_DSC_ = 0.3283 kcal/molK and Δ*S*_DSC_ = 0.313 kcal/molK, respectively. The ratio Δ*H*_DSC_/Δ*S*_DSC_ is 277 K = 4 °C for the disorder → order transition and 333 K = 60 °C for heat denaturation, in agreement with the heat capacity maxima.

The blue data points in Figure 3A are integrated with eqs 2–4 result in the black data points in panels 3B–3D. The comparison with cold denaturation in Figure 2 suggests a shift of the enthalpy by −78.3 kcal/mol, the enthalpy of cold denaturation. This scale shift (Figure 3B, red data points) leads to a zero enthalpy for the native protein and makes Figure 3 consistent with Figure 2. Likewise, the entropy in Figure 3C is shifted by −0.283 kcal/molK. The entropy of the native protein is now also zero. With these scale shifts the recalculated free energy is given by the red data points in Figure 3D. The free energy shows a trapezoidal temperature profile.

Figure 3A is almost a quantitative mirror image of cold-denaturation in Figure 2A. Not surprisingly, the red data points in Figure 3 related to cold denaturation are consistent with the direct measurements in Figure 2.

The experimental thermodynamic data for β-lactoglobulin are summarized in Table 1B.

### Analysis of DSC Thermograms with Three Different Models

#### Lysozyme Heat Unfolding

**Figure 4:**
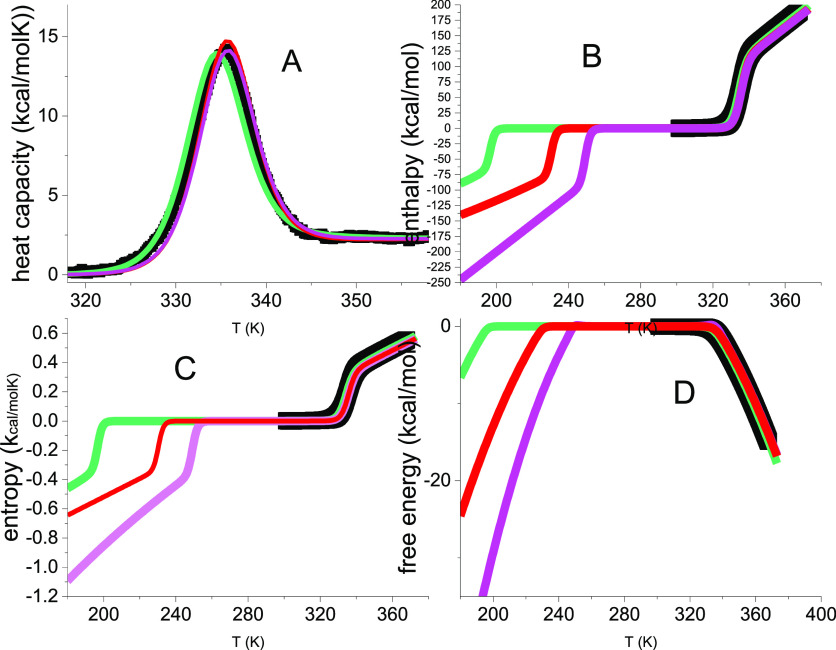
Analysis of lysozyme unfolding with three different models. (■) Experimental DSC results. Same data as in Figure 1. Magenta lines: Θ_U_(*T*)-weighted chemical equilibrium two-state model. Δ*H*_0_ = 107 kcal/mol, Δ*C*_p_^0^ = 2.27 kcal/molK. Red lines: Statistical-mechanical two-state model. Δ*E*_0_ = 110 kcal/mol, *C*_v_ = 1.05 kcal/molK. Green lines: multistate cooperative model. *h*_0_ = 0.90 kcal/mol, *c*_v_ = 7 cal/molK, σ = 5 × 10^–7^, *N* = 129. (A) Heat capacity. (B) Inner energy Δ*E*(*T*) (green, red), enthalpy Δ*H*(*T*) (magenta). (C) Entropy Δ*S*_v_(*T*), Δ*S*_p_(*T*). (D) Free energy Δ*F*(*T*), Δ*G*(*T*).

Figure 4 compares the experimental data of Figure 1 with the Θ_U_(*T*)-weighted chemical equilibrium model (magenta lines), the statistical-mechanical two-state model (red lines), and the multistate cooperative model (green lines). The simulations cover a large temperature range, predicting both heat and cold denaturation. However, no experimental data are available for lysozyme cold denaturation. Figure 4A shows virtually identical simulations of the heat capacity by the three models. The conformational parameters of the two-state models are almost identical (Δ*H*_0_ = 107 kcal/mol, Δ*E*_0_ = 110 kcal/mol). The simulation parameters are listed in Table 1B,C for the two-state models and in Table 2 for the multistate cooperative model.

**Table 2:** Thermodynamic Parameters of the Multistate Cooperative Model^16^

parameters	lysozyme	lactoglobulin Figure 6	lactoglobulin/1st peak Figure 7	lactoglobulin/2nd peak Figure 7
*h*_0_ (kcal/mol)^a^	0.91	–0.36	0.58	0.58
*c*_v_ (cal/molK)^b^	7	4	17.5	17.5
σ^c^	5 × 10^–7^	8 × 10^–5^	7 × 10^–5^	7 × 10^–5^
*N*^d^	129	160	80	80
Δ*E* (kcal/mol)^e^	136.2	–75.5	–76.57	112
Δ*S* (kcal/molK)^f^	0.406	–0.272	–0.277	0.336
Δ*F* (kcal/mol)^g^	–4.38	–5.2	–2.76	–4.68
Δ*S*_DSC_ (kcal/molK)^h^	0.417	–0.248	–0.282	0.313
*T*_m_°C (K)^i^	72(335)	5(278)	4(277)	58(331)
Δ*E*/Δ*S* °C (K)^j^	72 (335) °C	4 (277)	3(276)	60 (333)

a Unfolding enthalpy per amino acid residue.

b Molar heat capacity per amino acid residue.

c Cooperativity parameter.

d Number of amino acid residues participating in the unfolding transition.

e Predicted change in the inner energy.

f Predicted change in the unfolding entropy.

g Predicted change in the free energy.

h Entropy change, determined model-independently from DSC data.

i Midpoint temperature as determined by DSC.

j Predicted midpoint temperature from the changes of inner energy and entropy.

The three models provide good fits of all experimental thermodynamic properties (Figure 4B–D), predicting sigmoidal temperature profiles for enthalpy and entropy and a trapezoidal shape for the free energy. The models show differences with respect to cold denaturation. The statistical-mechanical models predict cold denaturation 20–50 °C lower than the Θ_U_(*T*)-weighted chemical equilibrium two-state model (cf. Figure 4B,C).

**Figure 5:**
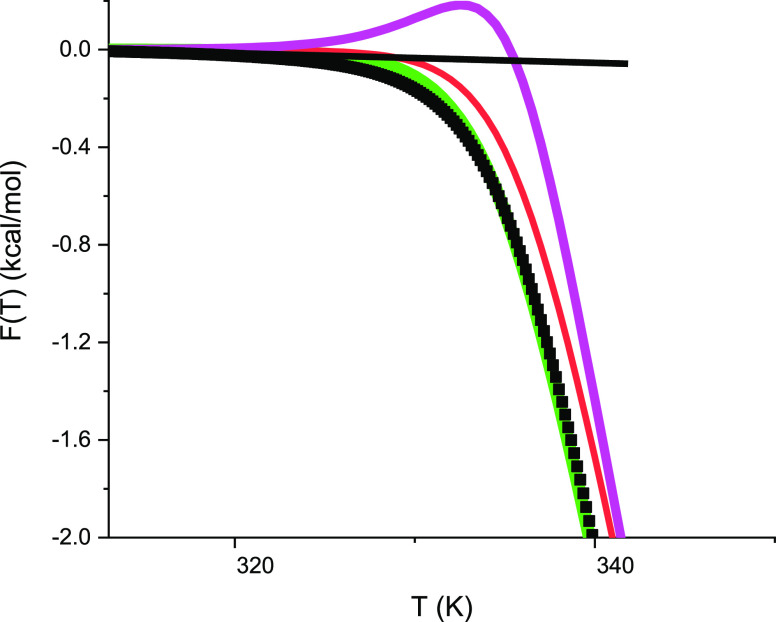
High-resolution temperature profile of the free energy. Black data points: same experimental results for lysozyme as in Figures 1 and 3. Magenta line: Θ_U_(*T*)-weighted chemical equilibrium model (eq 14, Δ*H*_0_ = 107 kcal/mol, Δ*C*_p_^0^ = 2.27 kcal/molK). Red line: statistical mechanical two-state model (Δ*E*_0_ = 110 kcal/mol, *C*_v_ = 1.05 kcal/molK; eq 16). Green line: multistate cooperative model. (*h*_0_ = 0.9 kcal/mol, *c*_v_ = 7 cal/molK, σ = 5 × 10^–7^, *N* = 129).

A further difference between the three models is shown in Figure 5, displaying the free energy at enhanced resolution. The DSC experiment reports a zero free energy for the native lysozyme, which becomes immediately negative upon unfolding. Of note, the experimental free energy is always negative, never positive. Both statistical-mechanical models reproduce this result correctly. In contrast, the Θ_U_(*T*)-weighted chemical equilibrium model displays a small positive peak in the vicinity of *T*_m_. Consequently, the free energies at the midpoint of unfolding are also different. The experimental free energy at *T*_m_ = 62 °C is Δ*H*(*T*_m_)_DSC_ = −0.76 kcal/mol. The multistate cooperative model predicts correctly Δ*F*(*T*_m_) = −0.73 kcal/mol and the statistical-mechanical two-state model Δ*F*(*T*_m_) = – *RT*_m_ ln 2 = −0.46 kcal/mol. In contrast, the Θ_U_-weighted chemical equilibrium two-state model yields exactly Δ*G*_Θ_(*T*_m_) = 0 kcal/mol. At *T*_m_ all three models predict the extent of unfolding as Θ_U_(*T*_m_) = 1/2. The protein is partially denatured at *T*_m_ and its free energy is necessarily negative.

**Figure 6:**
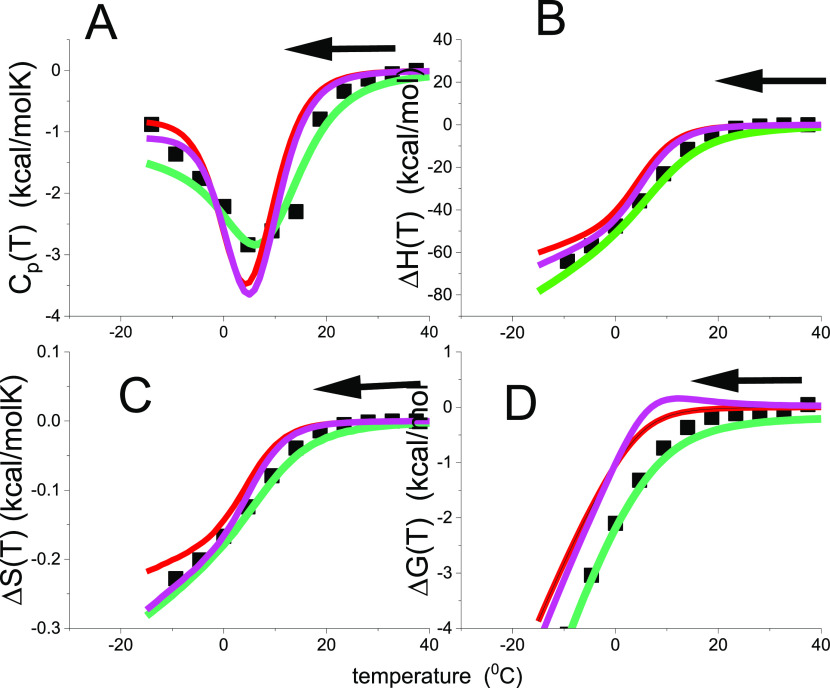
Cold denaturation of β-lactoglobulin. Same experimental data as in Figure 2 Arrows indicate the direction of cooling. The DSC-measurement starts with the native protein at 35 °C and decreases to −14 °C. Simulations with 3 different models. Magenta lines: Θ_U_(*T*)-weighted chemical equilibrium two-state model. Δ*H*_0_ = −42 kcal/mol. Δ*C*_p_^0^ = 1.1 kcal/molK, Red lines: statistical-mechanical two-state model. Δ*E*_0_ = −42 kcal/mol, *C*_v_ = 0.45 kcal/molK. Green lines: multistate cooperative model. *h*_0_ = −360 cal/mol, *c*_v_ = 4.0 caLL/mol, σ = 8 × 10^–5^, *N* = 160. (A) Heat capacity. (B) Enthalpy/inner energy. (C) Entropy. (D) Free energy.

The parabolic profile of the Gibbs free energy, which is predicted by the standard chemical equilibrium two-state model (eq 9), deviates even more from the DSC result and is hence not included in Figures 4D–6D.

#### β-Lactoglobulin. Cold Denaturation Analyzed with Different Models

Cold denaturation is analyzed with three different models. All models provide good fits of the thermodynamic properties. However, the Θ_U_(*T*)-weighted chemical equilibrium two-state model predicts some positive free energy, which is not supported by the DSC experiment. The multistate cooperative model provides the best simulation.

#### β-Lactoglobulin. Heat-Induced Folding and Unfolding Analyzed with Different Models

**Figure 7:**
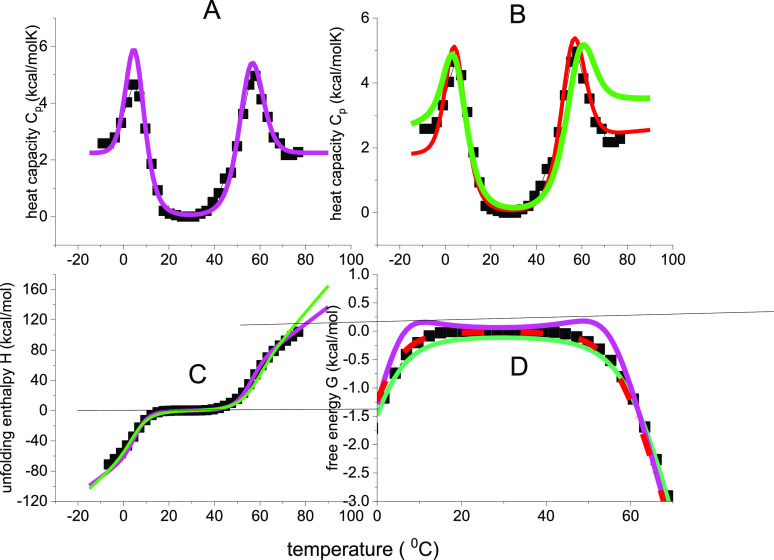
Heat-induced folding (at 4 °C) and unfolding (at 57 °C) of β-lactoglobulin in 2.0 M urea solution. DSC heat capacity data (black squares in panels A and B) are taken from ref (41). Black data points in panels C and D correspond to the red data points in Figure 3B,D. Magenta lines: Θ_U_(*T*)-weighted chemical equilibrium two-state model. Δ*H*_0_ = 56 kcal/mol; Δ*C*_p_^0^ = 2.25 kcal/molK. Red lines: statistical-mechanical two-state model. Δ*E*_0_ = 55 kcal/mol; *C*_v_ = 1.15 kcal/molK. Green lines: multistate cooperative model. *h*_0_ = 0.58 kcal/mol, *c*_v_ = 17 cal/molK, σ = 7 × 10^–5^, *N* = 80.

The simultaneous analysis of two heat-induced transitions is shown in Figure 7A for the Θ_U_(*T*)-weighted chemical equilibrium model (eq 11) and in Figure 7B for the statistical-mechanical models. All three models describe the temperature-profile of the heat capacity *C*_p_(*T*) equally well.

A criterion for protein stability is the temperature difference between heat and cold denaturation. DSC yields a temperature difference of Δ*T* = 53 °C between the heat capacity maxima. The prediction of the Θ_U_(*T*)-weighted chemical equilibrium model is  = 45 °C, that of the statistical-mechanical two-state model Δ*T* = Δ*E*_0_/*C*_v_ = 46 °C, and that of the multistate cooperative model Δ*T* ≈ *h*_0_/*c*_v_ = 48 °C.

The simulations of the three models overlap almost completely for heat capacity *C*_p_(*T*) and enthalpy Δ*H*(*T*)_DSC_ (Figure 7C). In contrast, the free energy prediction of the Θ_U_(*T*)-weighted chemical equilibrium model deviates from the experimental result in the vicinity of the phase transitions (Figure 7D). The DSC-derived free energy is zero or negative, never positive. The small positive peaks of the Θ_U_(*T*)-weighted chemical equilibrium two-state model disagree with this experimental result.

The total enthalpy of heat unfolding at 57 °C is Δ*H*_DSC_ = 104 kcal/mol, but the conformational enthalpy is only Δ*H*_0_ = 5 6 kcal/mol. The large difference is presumably caused by the binding of urea molecules and is Δ*H*_Δ*C*_p_^0^_ ∼ 50 kcal/mol.

The thermodynamic data and the fit parameters for β-lactoglobulin are summarized in Table 1B,C for the two-state models and in Table 2 for the multistate cooperative model.

## Discussion

### Model-Independent Analysis of DSC Experiments

The DSC experiment shows peaks of the heat capacity *C*_p_(*T*) at the temperatures of heat and cold unfolding. No folding model is needed to deduce the thermodynamic properties Δ*H*_DSC_(*T*), Δ*S*_DSC_(*T*), and Δ*G*_DSC_(*T*). The experimental results show sigmoidal curves for enthalpy and entropy and a trapezoidal temperature profile for the free energy. Different unfolding models can then be compared with the experimental data. Of note, the simulation must include not only the heat capacity, but also all three thermodynamic functions. This is ignored in the relevant literature.

### Spectroscopy and the Chemical Equilibrium Two-Stat Model

The chemical equilibrium two-state model is the almost exclusive model to fit spectroscopic unfolding transitions. Recent examples are found for nuclear magnetic resonance (NMR),^42,43^ CD,^1,44^ fluorescence,^45^ Raman spectroscopy,^46^ and elastic neutron scattering.^47,48^ Spectroscopic methods report structural changes, which only indirectly reflect thermodynamic changes. Indeed, a detailed comparison of CD spectroscopy and DSC for 10 different proteins revealed considerable differences between the van’t Hoff enthalpy of spectroscopy and the calorimetric unfolding enthalpy. The van’t Hoff enthalpy Δ*H*_0_ derived with eq 10 was typically 20–50% smaller than the calorimetric Δ*H*_DSC_ (see ref (1), Table 2). The analysis of the spectroscopic experiment becomes even more ambiguous if heat and cold denaturation are reported in the same experiment. This is illustrated for an NMR experiment with frataxin^43,49^ in the Supporting Information. Correct thermodynamic conclusions can only be made by comparison to DSC experiments.

Two-state models are simple approximations to cooperative protein unfolding. A large ensemble of micro-states is replaced by just two macro-states. The native and the unfolded protein conformation are assumed to be separated by a high free energy barrier and intermediate conformations are not populated. Intuitively, a two-state model is considered as the most cooperative limit of protein unfolding. However, it should be realized that the formalism of two-state unfolding contains no element of molecular cooperative interactions. Indeed, the statistical-mechanical two-state model follows from the cooperative multi-state model in the limit of no cooperativity.^26^

### Θ_U_(*T*)-Weighted Chemical Equilibrium Two-State Model

The standard model (eqs 7–11) correctly simulates the heat capacity *C*_p_(*T*), but fails for enthalpy, entropy and free energy. This is corrected here by multiplying the thermodynamic functions with the extent of unfolding Θ_U_(*T*), leading to the Θ_U_(*T*)-weighted functions 11–14. These thermodynamic relations simulate all experimental data quite well (magenta lines in Figures 2 and 5). In particular, the parabolic free energy of the standard chemical equilibrium model (eq 9) is replaced by a trapezoidal temperature profile (eq 14). However, as shown in Figures 4–7, the agreement between DSC and the Θ_U_(*T*) - weighted chemical equilibrium model is not perfect. The model predicts small positive free energies in the vicinity of the midpoints of unfolding, which is not supported by the experimental data.

### Statistical-Mechanical Two-State Model

The DSC experiment is intimately related to the protein partition function.^4,9,25,28,50,51^ The partition function *Z*(*T*) (eq 19) describes all thermodynamic properties. *Z*(*T*) follows from the Ising model^24^ as modified in ref (4, 25). The inner energy Δ*E*_0_ of the statistical-mechanical two-state model is almost identical to the conformational enthalpy Δ*H*_0_ of the chemical equilibrium model. However, no assumption about the entropy is required, which is in contrast to the chemical equilibrium two-state model (eq 8).^26^ The statistical-mechanical two-state model predicts a trapezoidal temperature profile of the free energy, which is in excellent agreement with the DSC experiments. The free energy is zero or negative, never positive. The molecular multistate partition function (eq 22) reduces to eq 19 if the cooperativity parameter is σ = 1 (= no cooperativity).^26^

### Multistate Cooperative Model^16^

The model is based on molecular parameters only. The unfolding enthalpy per amino acid residue is typically *h*_0_ ∼ 0.9–1.3 kcal/mol.^1^ This is confirmed by lysozyme with *h*_0_ = 0.9 kcal/mol. In contrast, β-lactoglobulin has low *h*_0_-values of 0.38–0.58 kcal/mol, probably caused by the high content of β-structure (cf. ref (52)). Multiplying *h*_0_ with the number of unfolded amino acid residues *n* yields an approximate conformational enthalpy Δ*H*_0_ = 60.8 kcal/mol.

Protein unfolding is a dynamic equilibrium of many short-lived intermediates, the probability of which is determined by the cooperativity parameter σ. Lysozyme unfolding is highly cooperative with a correspondingly small σ = 5 × 10^–7^. The probability of intermediates is distinctly reduced and lysozyme is the classical example for an apparent two-state unfolder. The cooperativity parameter σ is a physically well-defined quantitative measure of cooperativity (see below).

### Protein Stability and Free Energy

The basic tenet in protein folding is the assumption that proteins spontaneously fold into their native conformation. In the folding funnel hypothesis, the native proteins sit in a free energy minimum at the bottom of a rough-walled funnel. The folding process is a balanced enthalpy-entropy compensation. It involves a reduction in conformational entropy compensated by a gain in inner energy, resulting in a minimal free energy in favor of the folded structure. The common range of this minimal free energy that is quoted in the literature is 5–15 kcal/mol.^40^ The folding funnel is rather shallow^53,54^ and because of their small free energies of unfolding, proteins are often said to be only “marginally stable.”^55^

However, the free energy may not be the best criterion to judge protein stability. The trapezoidal free energy profile of β-lactoglobulin (Figures 3D and 7D) resembles an inverted “funnel.” The free energy change of the urea-destabilized protein is −3 kcal/mol at 4 °C and −4.35 kcal/mol at 57 °C. Interestingly, the free energy change of the more stable globular lysozyme is almost identical with −4.27 kcal/mol at 72 °C. The free energy allows no differentiation in the stability of the two proteins.

Alternative parameters may be better suited for defining stability. First, and most important is the midpoint temperature of heat unfolding *T*_m_. DSC measures directly and independent of any folding model, the unfolding enthalpy ΔH_DSC_ and the unfolding entropy Δ*S*_DSC_. The ratio of these thermodynamic parameters defines the midpoint temperature *T*_m_ assuming a first-order phase transition
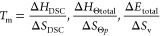
27

Table 1 shows the excellent agreement between the measured *T*_m_ and the predictions according to eq 27. A large unfolding enthalpy and a small entropy shift *T*_m_ to high temperatures. Equation 27 is equally applicable to *T*_cold_ as demonstrated for cold denaturation of β-lactoglobulin (cf. Table 1). Upon cold denaturation, the unfolding enthalpy of β-lactoglobulin is reduced by 30%, but the entropy by only 10%. The combined effect of these rather small changes is a reduction in unfolding temperature by 54 °C.

A second stability criterion is the temperature difference between heat and cold denaturation.^55^ The DSC experiment reveals a trapezoidal temperature profile of the free energy (Figures 3D and 5D). The temperature difference between heat and cold denaturation, Δ*T* = *T*_m_ – *T*_cold_, can be measured under favorable circumstances, but is usually not available experimentally. However, the Θ_U_(*T*)-weighted chemical equilibrium model predicts , the statistical-mechanical two-state model , and the multistate cooperative model . In all models the temperature difference ΔT increases with the conformational enthalpy Δ*H*_0_, inner energy Δ*E*_0_ and *h*_0_, and decreases with increasing heat capacities Δ*C*_p_^0^, *C*_v_, and *c*_v_. A large heat capacity corresponds to large energy fluctuations (eq 18), reducing the protein stability.

A third stability parameter is the width of the heat capacity peak itself. This is ∼28 °C for lysozyme and 43 °C for urea-destabilized β-lactoglobulin. The width of the transition peak reflects the strength of the intramolecular interactions and, in turn, the cooperativity of the system. A broad peak corresponds to a low cooperativity and a loser protein structure, whereas a sharp peak indicates a very cooperative system. A quantitative measure is the cooperativity parameter σ. The free energy to start a new folded sequence within an unfolded domain (nucleation) is given by Δ*G*_σ_ = – *RT* ln σ. For lysozyme (σ = 5 × 10^–7^) ΔG_σ_ is 9.6 kcal/mol, for β-lactoglobulin (σ = 7 × 10^–5^) the nucleation energy is 6.2 kcal/mol. These are large barriers for the initiation of new structures. The larger the nucleation energy, the more stable is the protein. The two proteins have almost identical free energies of unfolding, but their nucleation energies differ by 3.2 kcal/mol in favor of the more stable lysozyme.

Similar large free energies of structure initiation have been found in molecular dynamics calculations.^56,57^ The last comparison shows that the model-free analysis of thermodynamic unfolding data is not only important to test simple models but may also applied to the more advanced molecular dynamics results as, for example, described in the “dynameonics entropy dictionary.”^39^

## Concluding Remarks

The important thermodynamic properties for protein unfolding are enthalpy, entropy and free energy. These parameters can be obtained by measuring the heat capacity with differential scanning calorimetry, followed by integration of the thermograms. No unfolding model is needed. Rather on the contrary, the experimental temperature profiles Δ*H*(*T*)_DSC_, *T*Δ*S*(*T*)_DSC_, and Δ*G*(*T*)_DSC_ are necessary to test unfolding models, be it two-state unfolding or multistate cooperative unfolding. DSC experiments of lysozyme and β-lactoglobulin are presented. Enthalpy and entropy display sigmoidal temperature profiles while the free energy has a trapezoidal shape as observed experimentally for β-lactoglobulin. The experimental results are analyzed with two new two-state models, the Θ_U_(*T*)-weighted chemical equilibrium model and the statistical-mechanical model, and a multistate cooperative model. The standard chemical equilibrium model with its parabolic free energy profile does not fit the experimental data. Two-state models are suited for small proteins and provide macroscopic thermodynamic parameters. Molecular insight is gained only by applying a multistate cooperative model.

## Supporting Information Available

The supporting information compares the interpretation of the spectroscopic protein unfolding experiment with two different two-state models (PDF)
