# Supplementary material for: Protein Stability—Analysis of Heat and Cold Denaturation without and with Unfolding Models
Source: J Phys Chem B. 2023 Apr 11;127(15):3352–63. doi: 10.1021/acs.jpcb.3c00882 (PMC10123674; doi:10.1021/acs.jpcb.3c00882)
Supplement: Supplementary file 1 — jp3c00882_si_001.pdf [file jp3c00882_si_001.pdf]

Protein Stability - Analysis of Heat and Cold Denaturation without and with Unfolding Models

Joachim Seelig*‡ and Anna Seelig

‡Biozentrum, University of Basel, Spitalstrasse 41, CH-4056 Basel, Switzerland

*To whom correspondence should be addressed

Tel: +41-61 207 2190; e-mail: joachim.seelig@unibas.ch

Spectroscopic studies of heat and cold denaturation of yeast frataxin Yfh1

The vast majority of protein unfolding studies is performed with spectroscopic techniques (CD-, UV-, fluorescence-, IR-, Raman-spectroscopy, NMR). Differential scanning calorimetry (DSC) is less common. For 10 different proteins a detailed comparison of heat unfolding measured with spectroscopy and DSC was made[1]. Spectroscopy and DSC come to different thermodynamic conclusions. The spectroscopic transition is broader (less cooperative) than the DSC transition. More important, the unfolding enthalpy deduced from spectroscopy is usually only half of the calorimetric result.[1]

The analysis of spectroscopic data becomes even more confusing if cold denaturation is also included. A typical example is yeast frataxin, a protein with 174 amino acid residues,[2-3]

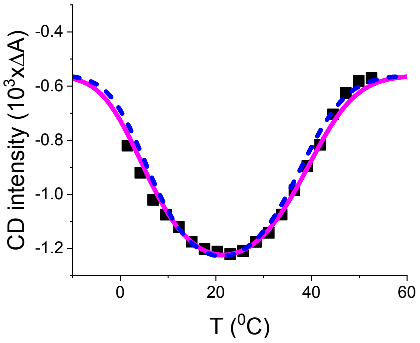

Figure S1. *Far UV absorbance of the CD spectra of Yfh1 in 20 mM Tris at pH 7.5. Black squares: experimental data taken from reference[2]. Blue line: chemical equilibrium two-state model (ΔH_0 = 21.4 kcal/mol ΔC_p^0 =1.81 kcal/molK,). Magenta line: statistical- mechanical two-state model (ΔE_0 = 20 kcal/mol, C_v = 0.82 kcal/molK), A_{min} = -1.56 , A_{max} = -0.57 for both models.*

The experimental data shown in figure S1 are obtained with CD spectroscopy and are taken from reference[2]. The authors confirm their CD temperature profile with NMR[2]. In a later publication the same data are discussed extensively in terms of the parabolic free energy profile of the chemical equilibrium two-state model.[3]

Figure S1 provides spectroscopic intensities, which are not identical with the fraction of unfolded protein Θ_U. The minimum and maximum of the transition curve are not necessarily $\Theta_U = 0$ and $\Theta_U = 1$. The interpretation of the intensity data requires the use of models. As is demonstrated in the following, quite different solutions are possible.

The simulation with the chemical equilibrium two-state model (blue line in fig. 1) uses the parameters of reference[2] (ΔH_{vH}^0 = 21.4 kcal/mol, ΔC_p^0 = 1.81 kcal/molK). The model then predicts the minimum extent of unfolding as Θ_U = 0.33 at 21°C. Yeast frataxin Yfh1 is

calculated to be 33% unfolded at 21 °C. The statistical-mechanical two-state model (magenta line) also provides an excellent fit (ΔE_0 = 20 kcal/mol, C_v = 0.82 kcal/molK). The calculated extent of unfolding is Θ_U = 0.3 at 21°C.

The thermodynamic consequences of the two models are, however, quite different and are illustrated in Figure S2.

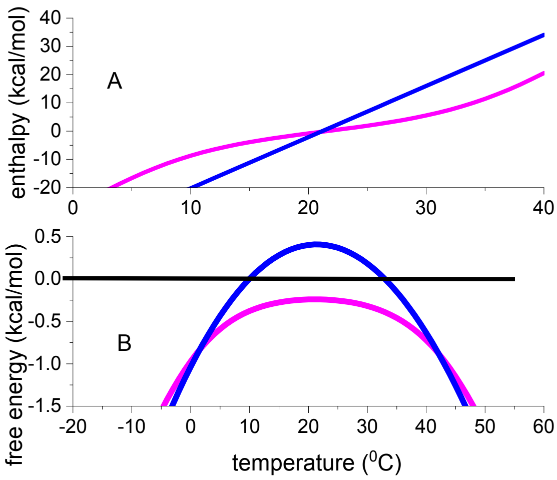

Figure S2. *Thermodynamic properties of Yfh1 (same parameters as in legend to fig. 1). Magenta lines: statistical-mechanical two-state model. Blue lines: chemical 2-state model. (A) Enthalpy (B) Free energy of unfolding*

The predicted unfolding enthalpies are linear in temperature for the chemical equilibrium two-state model and sigmoidal for the statistical-mechanical two-state model. The total unfolding enthalpies (temperature range 21 °C-55 °C) are 61.5 kcal/mol for the chemical equilibrium two-state model and 55.6 kcal/mol for the statistical-mechanical two-state model. These unfolding enthalpies are quite small for a protein with 174 aa.[1]

Figure S2B displays the free energies of unfolding. The statistical-mechanical two-state model predicts a trapezoidal shape with a minimum of -0.28 kcal/mol at 21 °C. In contrast, the

chemical equilibrium two-state model shows a parabolic shape with a positive maximum of 0.4 kcal/mol at 21 °C.

Figures S1 and S2 lead to the following conclusions. The primary spectroscopic data can be interpreted equally well with different models. The thermodynamic consequences are however quite different. On the basis of spectroscopy alone, no decision between the different models can be made. The model independent results of calorimetry suggest that only the statistical-mechanical two-state model predict the correct temperature profiles of the thermodynamic properties.

The calculations in figure S1 and S2 were made with parameters of reference[2]. The results make little sense in a broader context. An excellent simulation of the same experimental data with more meaningful parameters is shown in figure S3. The temperature profiles of the free energy are shown in figure S4.

S

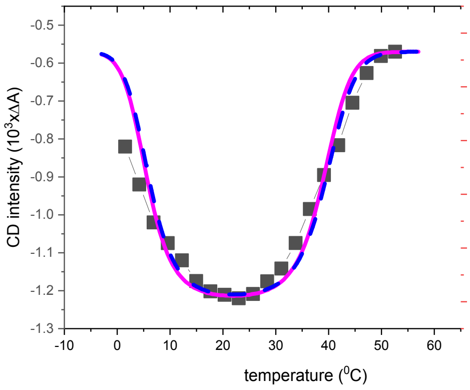

Figure S3. *Far UV absorbance of the CD spectra of Yfh1 in 20 mM Tris at pH 7.5.Black squares: experimental data taken from reference[2]. Magenta line: statistical-mechanical two-state model ($\Delta E_0 = 74$ kcal/mol, $C_v = 2.1$ kcal/mol). Blue line: chemical equilibrium two-state model ($\Delta H_0 = 65$ kcal/mol; $\Delta C_p^0 = 3.4$ kcal/mol K). $A_{min} = -1.21$, $A_{max} = -0.57$*

The fit parameters are larger by a factor 3-4 than those of figure S1 and are given in the legend to figure S3. Consequently, quite different thermodynamic properties are predicted. The fraction of unfolded protein at 21 °C is now calculated as Θ_U=0.03 by both models. The unfolding enthalpy is 139 kcal/mol for the statistical-mechanical two-state model and 135 kcal/mol for the chemical equilibrium two-state model. These large unfolding enthalpies are more plausible for a protein with 174 aa and compare well with other proteins.[1]

Figure S4 shows the temperature profiles of the free energy. The statistical-mechanical model displays a trapezoidal shape with a zero free energy for the native protein. In contrast, the chemical equilibrium two state model predicts a parabolic shape with a positive maximum of 1.9 kcal/mol for the native protein. A positive free energy for a stable protein has led to confusing discussions.[4-5]

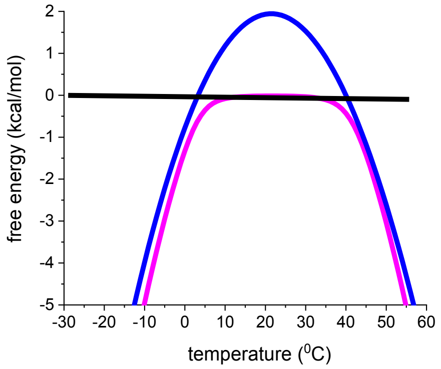

Figure S4*. Free energy of the chemical equilibrium two state model (blue line) and of the statistical-mechanical two-state model (magenta line). Calculations performed with the same parameters as listed in the legend of figure S3.*

General conclusions

Spectroscopic experiments provide no unique thermodynamic information. Different models can fit the experimental data. The statistical-mechanical two-state model is in agreement with general conclusions of calorimetry. Calorimetry does not support the positive maximum of the free energy as predicted by the chemical equilibrium two-state model. Spectroscopic data of unfolding can be calculated with a wide variety of parameters. A unique solution is not possible, unless a calorimetric measurement is performed.

References

1. Seelig, J.; Schönfeld, H.-J., Thermal protein unfolding by differential scanning calorimetry and circular dichroism spectroscopy two-state model versus sequential unfolding. *Quarterly Reviews of Biophysics* **2016,** *49*, e9 (24 pages).

2. Pastore, A.; Martin, S. R.; Politou, A.; Kondapalli, K. C.; Stemmler, T.; Temussi, P. A., Unbiased cold denaturation: Low- and high-temperature unfolding of yeast frataxin under physiological conditions. *Journal of the American Chemical Society* **2007,** *129* (17), 5374-+.

3. Sanfelice, D.; Morandi, E.; Pastore, A.; Niccolai, N.; Temussi, P. A., Cold denaturation unveiled: Molecular mechanism of the asymmetric unfolding of yeast frataxin. *Chemphyschem* **2015,** *16* (17), 3599-3602.

4. Becktel, W. J.; Schellman, J. A., Protein stability curves. *Biopolymers* **1987,** *26* (11), 1859-77.

5. Schellman, J. A., The thermodynamic stability of proteins. *Annu Rev Biophys Biophys Chem* **1987,** *16*, 115-37.
